# Copper metallurgical slag as a sustainable precursor of iron oxide photocatalysts to remove indigo carmine dye from water using the photo-Fenton process

**DOI:** 10.1007/s11356-025-36072-5

**Published:** 2025-02-19

**Authors:** Karen Valencia García, Melisa Portilla-Sangabriel, Agileo Hernández-Gordillo, Tania-Ariadna García-Mejía, Rosa-María Ramírez-Zamora

**Affiliations:** 1 Coordinación de Ingeniería Ambiental, Instituto de Ingeniería, Universidad Nacional Autónoma de México, Circuito Escolar S/N, Ciudad Universitaria, 04510 Coyoacán, Mexico City Mexico; 2 Instituto de Investigaciones en Materiales, Universidad Nacional Autónoma de México, Circuito Exterior S/N, Ciudad Universitaria, 04510 Coyoacán, Mexico City Mexico

**Keywords:** Advanced oxidation processes, Copper slag valorization, Maghemite nanoparticles synthesis, Water treatment, Photo-Fenton

## Abstract

The synthesis of an efficient composite photo-Fenton nanocatalyst was investigated using acid leaching processes of copper slag (*CS*) with two types of citric acid (EC = edible and AR = analytical-grade reagent). For this purpose, citric acid at room temperature or at 40 °C was used to lixiviate the elements from *CS*, and then, the leachates were subjected to a thermal treatment (350 °C). The formation of maghemite (γ-Fe_2_O_3_) accompanied with calcite as the main mineralogical phase was observed by X-ray diffraction (XRD) studies. The development of nanospheres dispersed in the calcite matrix and the proportion of γ-Fe_2_O_3_ in the catalyst or composite material were revealed by scanning electron microscope (SEM) and energy-dispersive X-ray spectroscopy (EDS), respectively. The catalyst performance in the Fenton and photo-Fenton processes was investigated in the degradation of the indigo carmine dye (*IC*, 10 ppm), achieving an efficiency of 96.1 ± 2.1% in 60 min, using 5 mM of H_2_O_2_ (30% weight) and UV-light (*λ* = 365 nm). The activity of these materials was analyzed and compared as a function of the specific surface area and the % γ-Fe_2_O_3_ in the composites. The preparation of maghemite-rich nanocomposite using the *CS* leachate, generated with edible citric acid, is an excellent alternative to substitute materials produced with analytical grade reagents because in the removal of *IC* dye, it showed similar efficiency, kinetics, and stability, but with lesser requirements of lamp energy radiation and doses of photocatalyst and oxidant, with respect to data reported in the literature.

**Supplementary Information:**

The online version contains supplementary material available at 10.1007/s11356-025-36072-5.

## Introduction

In the metallurgical industry, approximately 2.2 tons of copper slag (*CS*) are generated for every ton of copper produced from the pretreatment and refining of ores. Therefore, about 24.6 million tons of *CS* are produced each year and since the beginning of the industrial era. Current waste management options for *CS* include recycling, metal recovery, and slag landfill (Solís-López et al. 2014). The composition of *CS* depends on the type of furnace, the metallurgical production process, and the composition of the preceding minerals. Generally, *CS* is composed of Fe, Si, and Ca oxides with 35–60%, 25–40%, and 5–25% contents, respectively. Other oxides in lower percentages (< 10%) are also present in *CS* (Arzate-Salgado et al. 2016; Solís-López et al. 2014). Due to the chemical composition of *CS*, studies have been carried out in the field of environmental engineering using copper slags as (photo)-Fenton catalysts in the degradation of pollutants (Arzate-Salgado et al. 2016; García-Estrada et al. 2020; Herrera-Ibarra et al. 2022). However, they have shown a slow reaction speed due to the *CS* low specific surface area values of 0.68–16.00 m^2^ g^−1^ (García-Estrada et al. 2020; Huanosta-Gutiérrez et al. 2012; Solís-López et al. 2014).

The Fenton process is a homogeneous catalytic reaction, which at very low pH values uses Fe^2+^ ions to decompose hydrogen peroxide into hydroxyl radicals (•OH). These oxidizing species react with organic compounds, while Fe^2+^ is oxidized to Fe^3+^ ions. If the Fenton reagent is combined with UV–vis radiation (254 < *λ* < 600 nm), the reaction rate is accelerated and called the photo-Fenton process (Uma et al. 2020). Several variants have been developed to improve the efficiency of the degradation reaction and for avoiding a secondary contamination caused by iron acid sludge derived from the homogeneous Fenton process, among which the heterogeneous Fenton process stands out. This process is based on the use of insoluble or slightly soluble catalysts (instead of soluble Fe^2+^) that help to generate •OH radicals at circumneutral pH (Herrera-Ibarra et al. 2022). It is also possible to employ visible light or ultraviolet radiation to carry out the heterogeneous photo-Fenton process, allowing a considerable increase in the contaminant degradation reaction rate. For this purpose, the most commonly heterogeneous catalysts used in Fenton processes are iron oxides like Fe_3_O_4,_ α-Fe_2_O_3_, or $\gamma$-Fe_2_O_3_ since they are chemically stable materials, with catalytic activity at neutral pH (Ahmad et al. 2023; Cardona et al. 2023; Lei et al. 2021). Using these compounds, in the photo-Fenton process, various organic pollutants, such as pesticides (Varma et al. 2020), pharmaceuticals (Feng et al. 2023; Lu et al. 2023; Xu et al. 2022), and textile dyes (Lei et al. 2021; López et al. 2021; Yaou Balarabe et al. 2023), have been degraded by the heterogeneous photo-Fenton process.

Among these organic compounds, several dyes are discarded through effluents generated from the textile industry being this industry one of the primary sources of the water resources contamination, which has become a severe environmental problem worldwide (Juárez-Hernández et al. 2021; Lubis et al. 2019). One of the most common dyes used for dyeing denim is indigo carmine (*IC*), which is also used as a synthetic food coloring, but it becomes a toxic compound for humans when the concentration in wastewater exceeds 500 mg/kg body weight/day (Kuncser et al. 2022; Lei et al. 2021; Lubis et al. 2023). Various studies have managed to degrade the *IC* dye using iron oxides, hydrogen peroxide, and UV light. For example, Yaou Balarabe et al. (2023) synthesized a supported hematite (α-Fe_2_O_3_) catalyst by grinding Fe(NO_3_)_3_•9H_2_O as a precursor and then depositing it on a polyurethane membrane; through the photo-Fenton process using this supported catalyst, hydrogen peroxide and a 125 watts Mercury lamp (UV), they managed to reduce around 65% of *IC* initial concentration (25 ppm) in 35 min. On the other hand, Show et al. (2016) synthesized thin films of α-Fe_2_O_3_ nano-spheres by an electrochemical route using a chloride tetrahydrate (FeCl_2_•4H_2_O) solution as a precursor. Through the photocatalysis process using a 200 W tungsten lamp, they achieved to degrade 96% of *IC* (100 ppm) in 150 min. However, most of the reported iron oxide catalysts are produced using analytical-grade reagent precursors, which represent high costs. Further processing generally generates waste due to its manufacture, which is a major drawback for their use on an industrial scale. Therefore, an alternative to prepare iron oxide is using wastes or by-products with high iron content, such as copper metallurgical slag. Thus, copper slag (*CS*) is economically attractive as a precursor for iron oxide catalyst production because of its high iron content.

The possibility of recovering iron from discarded copper slag has been investigated through pyrometallurgical, hydrometallurgical, and acid leaching processes (Kundu et al. 2023), being the acid leaching-based process one of the most selective and efficient for metal extraction. Generally, sodium chlorate, aqueous sulfur dioxide, sulfuric acid, nitric acid, mixed nitric-sulfuric acid, or potassium dichromate are the potential lixiviants for the treatment of copper slag reported in the literature (Kundu et al. 2023; Wang et al. 2023, 2024a, b; Xiao-Dong et al. 2022). The main disadvantages of this process are the use of aggressive acids, long stirring times of up to 48 h, and high acid concentrations of up to 3 M (Wang et al. 2023, 2024a, b).

Citric acid is more widely available, economical, and environmentally friendly than harsh inorganic acids. This acid acts as a chelating agent and forms complexes such as the iron citrate complex (Mele et al. 2024). In this sense, this research aimed to assess the use of the copper slag (*CS*) leachate with citric acid as a source of iron oxide. Three variants of the leaching process were studied using two types of citric acid and two concentrations: (1) edible grade citric and (2) edible grade citric acid dissolved at 40 °C and (3) analytical-grade citric acid reagents. The performance of the synthesized materials was investigated in the degradation of *IC* dye by Fenton and photo-Fenton processes under UV-lamp irradiation (365 nm, 15 watts).

## Methodology

### Synthesis of composite materials using CS and citric acid

Iron catalysts were prepared from copper slag (*CS*) with citric acid leaching, varying the source and concentration of the citric. The *CS* sample was obtained from a copper smelting industry in northern Mexico. The particle size of the slag was $\sim$ 74 µm (200 mesh). For this purpose, 1 L of citric acid (C₆H₈O₇) solution was prepared at *X* = 0.2 or 0.4 M from analytical-grade reagents, Sigma-Aldrich 99% (AR), or Taste & Joy edible grade citric acid at room temperature (EC) or with the solution heated to 40 °C for completely dissolve the edible citric acid (DEC). Then, 40 g of *CS* was added to the citric acid dissolution maintained mechanically stirred with paddles at 300 rpm for 1 h. Subsequently, the solid residue (unleached slag) was separated by filtration, and the leachate was recovered. The recovered leachate was evaporated to dryness to obtain a powder, and then, it was subjected to thermal treatment at 350 °C for 2 h, with a heating ramp of 5 °C/min. The obtained powder samples were labeled according to the type grade reagents and the dissolution conditions as *X*-AR, *X*-EC, and *X*-DEC, where *X* represents the molarity of the citric acid solution.

### Characterization of CS and synthesized composite materials

The copper slag was characterized by X-ray fluorescence (XRF) using a Siemens SRS300 spectrometer, and the comparison was carried out with EZSCAN Rigaku Primus II. By X-ray diffraction (XRD) using an EMPYREAN diffractometer equipped with Ni filter, fine focus copper tube and PIXcel3D detector and a scanning range between 20 and 65° (2 theta) with a step size of 0.003°/s and an integration time of 40 s per step. Identification and quantification of mineralogical phases were carried out using the HIGHScore v4.5 software and the ICDD (International Center for Diffraction DATA) and ICSD (Inorganic Crystal Structure Database) databases. The specific surface area of the material was determined by the BET method from the nitrogen adsorption–desorption isotherms at 77 K. The analysis was carried out using a Bel-Japan Minisorp II instrument. Before measurements, the powdered samples were degassed at 200 °C in a vacuum for 12 h.

The synthesized materials were characterized by the X-ray diffraction (XRD) method previously described. The FTIR absorption spectra were recorded on a Thermoscientific Nicolet 6700 spectrometer provided with an ATR accessory and a diamond crystal. The pressure used was 815 Psi at room temperature and; 68 scans were done at a resolution of 4 cm^−1^ in the interval from 400 to 3500 cm^−1^ in the transmittance mode. The morphology of the samples was analyzed by scanning electron microscopy (SEM) using a JEOL 7800 F at 10 kV, equipped with an energy-dispersive X-ray analyzer (EDS) (S-3400 N) to identify and quantify the elements present in the sample. The high-resolution transmission electron microscopy (HRTEM) analysis was performed using a Jeol ARM200F Schottky field emission electron microscope with atomic resolution, equipped with a Spherical Aberration Corrector (Cs) in STEM mode that allows obtaining a resolution of 80 pm. High-angle annular dark field (HAADF) imaging allows for differentiating of elemental chemistry through the difference in contrast. The specific surface area of the material was determined using the previously described BET method. The UV–vis diffuse reflectance spectra (DRS) were obtained on a Shimadzu 2600 equipped with an integration sphere ISR 2600 in the interval from 200 to 800 nm BaSO_4_ which was used as a reference blank. The absorbance spectra were obtained, and the bandgap energy (*Eg*) was calculated using the Kubelka–Munk model, considering a direct transition ((F(R)x*hv*)^2^) for γ-Fe_2_O_3_ material (Cabrera et al. 2024).

### Degradation tests of indigo carmine dye using the synthesized composite materials

The synthesized materials (composite material) were evaluated in degradation experiments of *IC* dye in three heterogeneous advanced oxidation processes (AOP): photocatalysis, Fenton, and photo-Fenton methods, using the experimental conditions described in Table 1. Photo oxidation, oxidation, and photolysis trials were performed and used as blank tests.

**Table 1:** Experimental conditions of degradation tests of *IC* dye (250 mL of 10 ppm of *IC* dye)

Process	UV light (*λ* = 365 nm)	Composite material	H_2_O_2_ (5 mM)
Photolysis	X	–-	–-
Photocatalysis	X	X	–-
Oxidation	–-	–-	X
Photo-oxidation	X	–-	X
Fenton	–-	X	X
Photo-Fenton	X	X	X

The mass of the composite material was 25 mg in 250 mL, equivalent to 0.1 g/L

The methodology for the three AOPs was as follows: 25 mg of the composite material in powder was added to 250 mL (0.1 g/L) of an *IC* dye aqueous solution at 10 ppm at natural pH (~ 6.5) prepared with distilled water. Before irradiation, the suspension was stirred at 1000 rpm at room temperature (20–22 °C) in an open-air glass reactor system and was kept in dark conditions for 30 min (adsorption–desorption equilibrium). Subsequently, for the photocatalysis method, the suspension was irradiated during 60 min using a 15 W UV light (maximum emission light at *λ* = 365 nm) placed inside the glass reactor. For the Fenton methods, 5 mM (100 µL) of H_2_O_2_ (Meyer, 30% weight) was added at room temperature (20–22 °C), reaching a pH ~ 6.7, and for photo-Fenton, H_2_O_2_ with UV light irradiation was used. The methodology for the blank tests was similar to the AOP trials but without adding the catalyst. In all cases, the concentration of *IC* dye in a filtered aliquot of 3 mL collected at different time intervals (1, 3, 5, 10, 20, 40, and 60 min) was quantified by following the absorption band at 610 nm, using a UV–vis Shimadzu 1800 spectrometer. The *IC* removal rate (*r*) was fit to pseudo-first-order kinetics with an apparent rate constant (*K*_*app*_), where the reactant concentration after reaching equilibrium was taken as the initial concentration (*C*_0_), which is expressed as follows: *-ln(C/C*_0_*)* = *K*_*app*_*t*. Plotting *-ln(C/C*_*0*_*)* versus reaction time (*t*) yields a straight line, and the slope is the apparent kinetic rate constant (*K*_*app*_). The stability of the best sample was evaluated in 6 cycles of reaction using the same best condition. The total iron in the solution was measured using the standardized photometric method, Spectroquant Iron Testa (0.025–5.00 mg/L), with a Nova spectrometer.

### Determination of reactive oxygen species (ROS)

An indirect determination of hydroxyl radicals (•OH) was performed by applying salicylic acid dosimetry (Arzate Salgado et al. 2024; Milne et al. 2013), which is a method based on the principle of an aromatic hydroxylation of this compound using the sample 04DEC. The technique indicates that in the presence of •OH, salicylic acid turns into 2,3-dihidroxybenzoic acid (2,3-DHBA) and 2,5-dihidroxybenzoic acid (2,5-DHBA). The formation of these hydroxylated by-products indicates the generation of •OH radicals. In order to determine the production of the hydroxylated by-products, 250 mL of solution of salicylic acid (20 mg/L) in distilled water and 25 mg of 04DEC sample were added as well as hydrogen peroxide (5 mM) under the same conditions that were used in the Fenton and photo-Fenton processes as it was previously described above. The hydroxylated by-products were detected by using an 1100 Agilent chromatography apparatus equipped with a photodiode array detector (Agilent Technologies, Inc., Santa Clara, CA, USA). The stationary phase was an ACE5 C18-AR column, and the injection volume of the samples was 30 µL. The mobile phase was constituted by a mixture of methanol, acetonitrile, and phosphoric acid (0.1%, v/v) at a 10:8:82 ratio (v/v). The eluent flow rate was 1.0 mL/min during the first 11 min and then 1.5 min for the next 12 min. The detection wavelengths (*λ*) of each compound were the following: *λ*1: 236 nm for 2.5-DHBA and *λ*2: 246 nm to detect 2.3-DHBA. The retention times were 7 and 9 min, respectively. Also, another indirect determination of hydroxyl radicals (•OH) was also performed with the fluorescence of 2-hydroxyterephtalic acid (HTA) at 425 nm (Bernal-Díaz et al. 2024). For this purpose, 0.5 mM of terephthalic acid (TA) was prepared using an aqueous solution of NaOH 2 mM. Each sample was immersed in 250 mL of TA solution and left under dark conditions and constant stirring (1200 rpm) for 30 min. Then, the samples were illuminated using the same Fenton and photo-Fenton experimental conditions. The fluorescence was measured with an FS5 Edinburgh Instruments spectrofluorometer using an excitation wavelength of 312 nm. Alternatively, the presence of superoxide radicals (•${O}_{2}^{-}$) was evaluated indirectly (Bernal-Díaz et al. 2024). The IC degradation was evaluated using the same experimental conditions previously described for the Fenton and photo-Fenton processes under anaerobic conditions through the displacement of dissolved oxygen in the solution, by bubbling nitrogen during the evaluation of each process (Fenton and photo-Fenton). The technique indicates that if the concentration of dissolved oxygen decreases in the solution this prevents the production of •${O}_{2}^{-}$ radicals; then if the degradation of the *IC* dye was caused by •${O}_{2}^{-}$ radicals, then the *IC* degradation would be inhibited.

## Results and discussion

### Characterization of copper slag (CS)

**Fig. 1:**
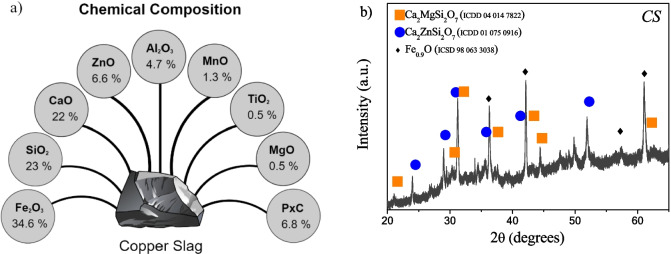
**a** Chemical composition of *CS* by XRF and **b** mineralogical phases by XRD

Figure 1 shows the scheme of percentual chemical composition for copper slag determined by X-ray fluorescence analysis, where the elements are written in terms of oxides by convention. The *CS* exhibits contents of 34.6% Fe_2_O_3_, 23.0% SiO_2_, and 22.0% CaO in major proportions, while the rest: ZnO, Al_2_O_3_, MnO, TiO_2_, and MgO are in proportions less than 6.6%. The relatively high iron content present in *CS* constitutes a source of iron to synthesize iron oxide catalyst materials. The XRD pattern for *CS* is shown in Fig. 1b, which exhibited reflection peaks that are indexed to akermanite Ca_2_MgSi_2_O_7_ (ICDD 04 014 7822), hardystonite Ca_2_ZnSi_2_O_7_ (ICDD 01 075 0916), and wuestite FeO (ICSD 98 063 3038). The presence of these phases in *CS* is consistent with the main components found in the X-ray fluorescence analysis and corresponds to the reported phases in this type of slag (Yañez-Aulestia and Ramírez-Zamora 2023). The textural properties of the *CS* were studied by N_2_ adsorption–desorption isotherms (Fig. S1), which exhibited a curve characteristic of type I isotherm with H4 type hysteresis loop. Type I isotherms are given by microporous solids having relatively small external surface area; type H4 loop is often associated with narrow slit-like pores, but in this case, the type I isotherm character is indicative of microporosity (Sing et al. 1985). In addition, the estimated specific surface area obtained for the *CS* is 0.7 m^2^ g^−1^, which confirms the microporosity.

### Crystalline structures of the composite materials

**Fig. 2:**
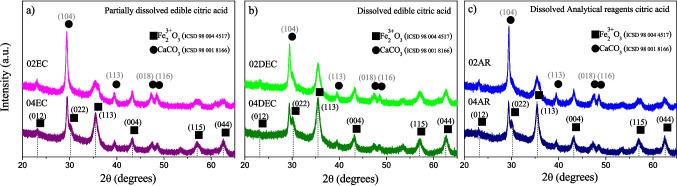
X-ray diffraction patterns of synthesized composite material prepared with different types of citric acid grade reagents: **a** EC, **b** DEC, and **c** AR at 0.2 and 0.4 M of citric acid

Figure 2a–c show the XRD patterns of the synthesized materials prepared by acid leaching processes with two types of citric acid, edible grade (EC and DEC) and analytical reagents (AR), at concentrations of 0.2 and 0.4 M of citric acid. All the samples exhibit the diffraction peaks at 23.5, 30.3, 35.6, 43.3, 57.2, and 62.9° in 2*θ*, corresponding to the (012), (022), (113), (004), (115), and (044) diffraction planes, respectively, which are associated with maghemite (γ-Fe_2_O_3_) (ICSD 98 004 4517). Additionally, the peaks located at 29.4, 39.4, 47.6, and 48.5° in 2*θ*, corresponding to the (104), (113), (018), and (116) diffraction planes, respectively, are associated with the calcite (CaCO_3_) (ICSD 98 001 8166). However, a slight variation was observed when diverse citric acid concentrations were used for the different leaching conditions. When dissolving edible or analytical citric acid at room temperature, it is observed that, at low citric acid, the 02AR and 02EC samples exhibit diffraction peaks linked to CaCO_3_, which is more intense than those maghemite peaks; whereas dissolving edible citric at 40 °C, the 02DEC samples slightly increased the intensity of maghemite peaks in the (113) planes, suggesting that dissolving completely the citric acid promotes the iron leaching. Subsequently, increasing the citric acid concentration at 0.4 M for the two leaching conditions, the three samples (EC, DEC, and AR samples) exhibited the most intense peaks associated with γ-Fe_2_O_3_. For the 02EC and 04EC samples, the CaCO_3_ content is in the intervals of 54.5, 39.2%, and the γ-Fe_2_O_3_ content is 45.5, 60.8%, respectively. Whereas for the 02DEC and 04DE, completely dissolving citric acid, the CaCO_3_ content is 34.8, 24.6%, and for γ-Fe_2_O_3_ is 65.2, 75.4%, respectively. When citric acid analytical-grade reagent is used, the CaCO_3_ content is 44.8, 28.8%, and the γ-Fe_2_O_3_ content is 55.2, 71.2%, respectively. The variability in the γ-Fe_2_O_3_ content of the *CS* leached is due to the difference in the type of citric acid grade reagents: when the edible grade is used at room temperature, the citric acid is partially dissolved in the solution, and therefore, minor iron content is complexed from *CS*. By contrast, when edible grade at 40 °C or analytical-grade reagent is used, a major amount of citric acid is completely dissolved in the solution, being capable of complex a greater amount of iron present in the *CS*.

### Surface analysis by FTIR spectroscopy of the composite materials

**Fig. 3:**
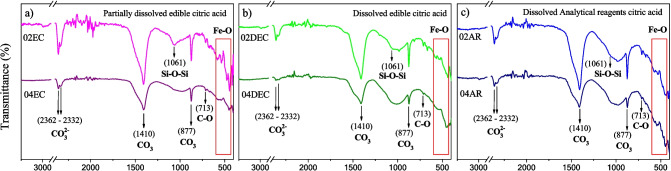
FTIR of the synthesized composite material prepared with different types of citric acid grade reagents: **a** EC, **b** DEC, and **c** AR at 0.2 and 0.4 M of citric acid

The FTIR patterns of all materials, prepared with different types of citric acid grade reagents at 0.2 and 0.4 M of citric acid, are shown in Fig. 3a–c. Each spectrum shows similar signals, suggesting that the bonding structures on the surface are similar among them. The observed absorption bands at 2362 and 2332 cm^−1^ belong to the stretching vibrations of the CO_3_^2−^ groups, and the bands at 1410, 877, and 713 cm^−1^ are characteristics of the antisymmetric stretching of CO_3_ and symmetrical stretching of CO, respectively (Hsiao et al. 2019). These intense bands are related to three types of C-O bond vibrations (v2, v3, and v4), characteristic of the calcite polymorph (CaCO_3_) present in the sample (Onimisi et al. 2016). Additionally, the absorption bands at 1061 cm^−1^ could be related to the antisymmetric stretching vibration peak of the Si–O-Si group, which belonged to the characteristic peak of SiO_2_ (Shi et al. 2022), since silicon may have been leached from the *CS*. Likewise, the characteristic bands of γ-Fe_2_O_3_ are also exhibited. The absorption bands at 663, 578, 528, and 444 cm^−1^ are associated with the vibrations of the Fe–O bonds (Cabrera et al. 2024; Zheng et al. 2024). However, it can be observed that when 0.2 M of citric acid is used for leaching (Fig. 3a–c), the absorption bands corresponding to the vibrational modes of polymorph CaCO_3_ are more intense than those obtained at 0.4 M of citric acid, following this behavior regardless the type of citric acid and the leaching conditions used. These results derived at a low concentration of 0.2 M can be explained in terms of the formation constant of calcium citrate (log *K*_1_ = 4.68), which is higher with respect to iron (II) citrate (log *K*_1_ = 3.08). At fewer molecules of citric acid, the leaching of calcium is more favored than that of iron, generating lower concentrations of iron citrate complex. Thus, when the concentration of citric acid is two-fold higher, there are enough molecules for dissolving iron, decreasing calcium percentage in the CS leachate. The presence of both CaCO_3_ and γ-Fe_2_O_3_ materials, as was observed by the XRD analysis, suggests the formation of a mixed composite type where CaCO_3_ will work as a dispersing matrix (Walsh et al. 2011).

### Morphology and textural properties of the synthesized composite materials

**Fig. 4:**
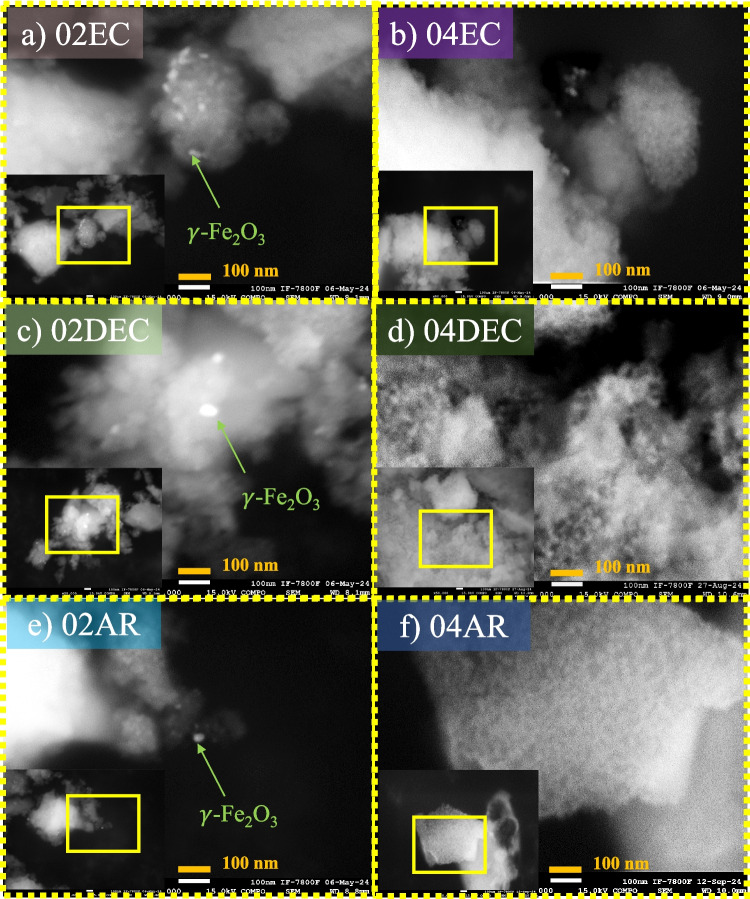
SEM image of the synthesized composite materials prepared with different types of citric acid grade reagents: **a** EC, **b** DEC, and **c** AR at 0.2 and 0.4 M of citric acid

The variations in the morphology of the composite materials prepared with different acid leaching conditions (type of citric acid grade at room temperature and 40 °C) are shown in Fig. 4a–f. In all three cases, the composite samples exhibit nanoparticles of sphere morphology with diameters ranging from 5 to ∼10 nm, which are immersed in a cotton-like mass of relatively large dimensions (100–500 nm). A very similar distribution and size of the nano-spheres is observed between the six catalysts (02EC, 04EC, 02DEC, 04DEC, 02AR, and 04AR), regardless of the type of citric acid used and the leaching conditions used for its preparation. Considering that the micrographs of samples were collected by means of backscattered electrons, where the light intensity varies proportionally with the atomic number of the elements, the observed brightness of the well-dispersed nano-spheres particles suggests that it is probably composed of γ-Fe_2_O_3_, whereas that rest of the low brightness in the cotton-like mass will be CaCO_3_, as was detected by FTIR analysis, functioning as the dispersing matrix.

**Fig. 5:**
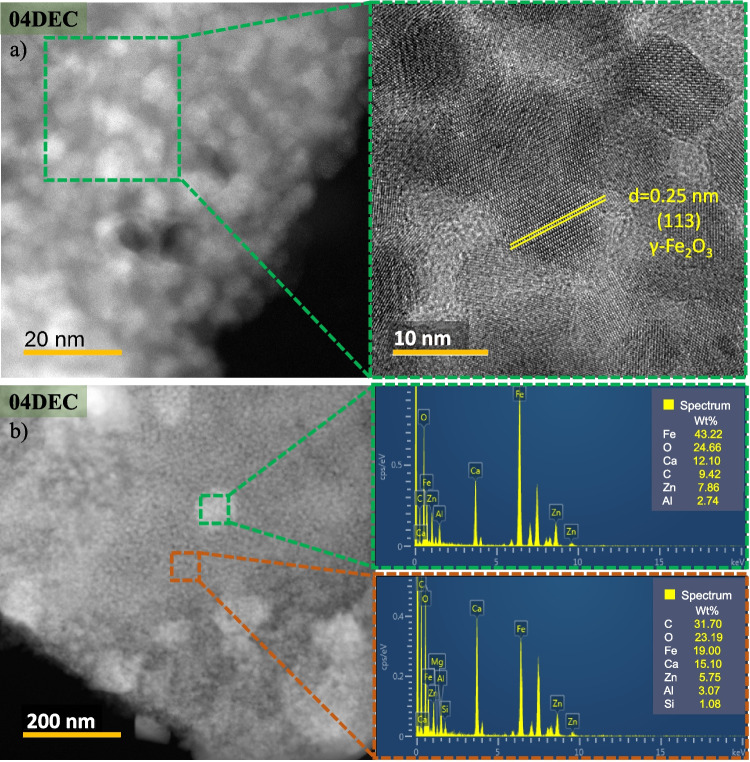
HRTEM **a** image and **b** EDS of the synthesized composite material 04DEC

The HRTEM images of the 04DEC sample, taken with backscattered electrons, are shown in Fig. 5a. It is observed that the composite sample is formed of nano-spheres with a size of about 10 nm. The measured lattice distance for the nano-sphere is 0.25 nm, which coincides with the interplanar distance of the (113) plane of the maghemite, being the main plane observed by XRD. The EDS spectra taken in two different regions are shown in Fig. 5b, where it is observed that in the brightest zone, the surface composition of major contents is 43.22, 24.66, and 12.10 wt.% is of Fe, O, and Ca, respectively, while that minor content of 9.42, 7.86, and 2.74 wt.% is of C, Zn, and Al, respectively. For the less bright area, the surface compositions of Fe and Ca are almost similar (19 and 15.10 wt.%). This suggests that the nano-spheres correspond to the γ-Fe_2_O_3_ phase, and other elements (Zn, Al, and Si) were detected in smaller proportion, which could have been leached from the *CS* during the acid leaching process. In addition, Zn could be present as zinc oxide since, approximately, at 350 °C takes place, the transformation of zinc citrate complex to zinc oxide (Xie et al. 2014). Also, Al could be found as $\gamma$-Al_2_O_3_ phase (López-Juárez et al. 2018), although it was not possible to detect it in the XRD analysis. On the other hand, the presence of silicon could be in oxide since, in FTIR, the absorption band associated with the Si–O-Si bonds was detected (1061 cm^−1^).

The textural properties of the composite materials were studied by N_2_ adsorption–desorption isotherms (Fig. S2). The 02EC, 02DEC, 04EC, and 04AR samples exhibit curves of the type IV isotherm, with the H1 hysteresis loop presenting a saturation at relative pressure close to 0.8–0.9. This type of hysteresis is commonly formed in materials that have a narrow distribution of uniform cylindrical mesopores (Xu et al. 2020), whereas the samples 02AR and 04DEC exhibit the isotherm curve type IV, with H3 hysteresis loop characteristic of mesoporous materials with wedge-shaped pores (Valencia et al. 2022). The estimated specific surface area (SA) values for the composites prepared with edible citric acid, dissolved at room temperature, were 73.5 and 76.8 m^2^ g^−1^ for the 02EC and 04EC, respectively. While for those prepared at 40 °C (02DEC and 04DEC), it is in the range of 93.4 to 121.7 m^2^ g^−1^ (Table 2), respectively. It can be observed that when the edible grade reagent is dissolved at room temperature, the composites exhibit a low SA, whereas dissolving citric acid at 40 °C, the 04EC sample presents the highest value of this parameter (121.7 m^2^ g^−1^). In some cases, the SA values of the composites are greater than data reported in the literature when the γ-Fe_2_O_3_ is prepared from analytical grade precursors (Bi et al. 2023; Mo et al. 2024; Wang et al. 2024a, b; Zheng et al. 2024). The high SA for 04AR and 04DEC samples can be explained by the formation of smaller nano-spheres particles dispersed into the CaCO_3_ matrix, where uniform cylindrical pores were formed. In the case of the 04DEC sample, wedge-shade pores could contribute to an increase in mesoporosity compared to composites with uniform cylindrical pores.

**Table 2:** Content of CaCO_3_ and γ-Fe_2_O_3_ present in each sample, specific surface area and band gap

Sample name	Contents from XRD			
	CaCO_3_ [%]	γ-Fe_2_O_3_ [%]	SA [m^2^ g^−1^]	Eg [eV]
Partially dissolved citric acid				
02EC	54.5	45.5	73.5	1.99
04EC	39.2	60.8	76.8	2.00
Dissolved citric acid				
02DEC	34.8	65.2	99.5	1.91
**04DEC**	**24.6**	**75.4**	**121.7**	**1.91**
02AR	44.8	55.2	97.7	1.98
04AR	28.8	71.2	93.4	1.98

The data in bold indicate the material with the greatest specific surface area and highest maghemite content

### Optical properties of the synthesized catalysts

**Fig. 6:**
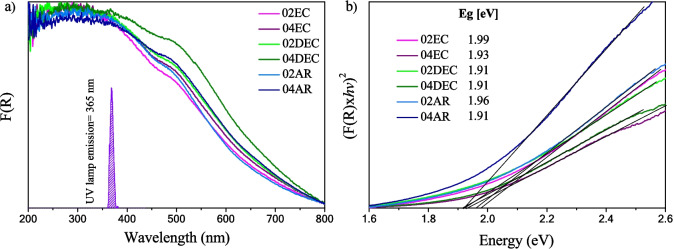
**a** UV–vis DRS spectra synthesized composite materials prepared with different types of citric acid grade reagents: **a** EC, **b** DEC, and **c** AR at 0.2 and 0.4 M of citric acid

The UV–vis diffuse reflectance spectra (DRS) of all the composite samples are shown in Fig. 6a. All materials begin to absorb around 800 nm, with a maximum absorption at approximately 280 nm, indicating that the absorption occurs within the UV–vis light region. It is known that the maximum absorption of γ-Fe_2_O_3_ is around 500 nm (Cabrera et al. 2024), while that of CaCO_3_ is at approximately 260 nm (Al Omari et al. 2016). However, nearby 500 nm, an absorption shoulder is increased in the following order 02EC $<$ 02AR $<$ 04EC $<$ 02DEC $<$ 04AR $<$ 04DEC sample. This variation may be related to the greater content of γ-Fe_2_O_3_. According to the emission spectrum of the UV lamp (inset in Fig. 6a), the γ-Fe_2_O_3_ present in all the composites can be activated by UV-C irradiation at 365 nm, whereas CaCO_3_ cannot be activated due to the large band gap energy of calcite, which is reported around 4.3 eV (Al Omari et al. 2016; Kaur and Singh 2023) (Zhou et al. 2025). The band gap energy value of all the composite solids was determined using the Kubelka–Munk function, assuming a direct transition of γ-Fe_2_O_3_ (Cabrera et al. 2024), given closed values in the interval of 1.95 ± 04 eV (Fig. 6b, Table 2). The band gap energy value agrees with that reported for γ-Fe_2_O_3_ of approximately 1.9 eV (Cabrera et al. 2024), (Al Omari et al. 2016; Kaur and Singh 2023). In addition, a slight blue-shifted in the optical absorption towards higher energies of nanocomposite materials was observed, being associated with the increase in the band gap energy values. This result can be related to the presence of a higher content of CaCO_3_, as was indicated for the 02EC sample by EDS (Table 2), which contains 54% of calcite.

### Performance of composites in the IC dye removal tests

The efficiency of the composite materials to degrade or discolor a 10 ppm of *IC* dye solution was evaluated in three heterogeneous processes: photocatalysis, Fenton, and photo-Fenton process (Table 1), and also using blank tests to estimate the catalyst contribution to degrade IC.

#### Processes of low degradation efficiency

**Fig. 7:**
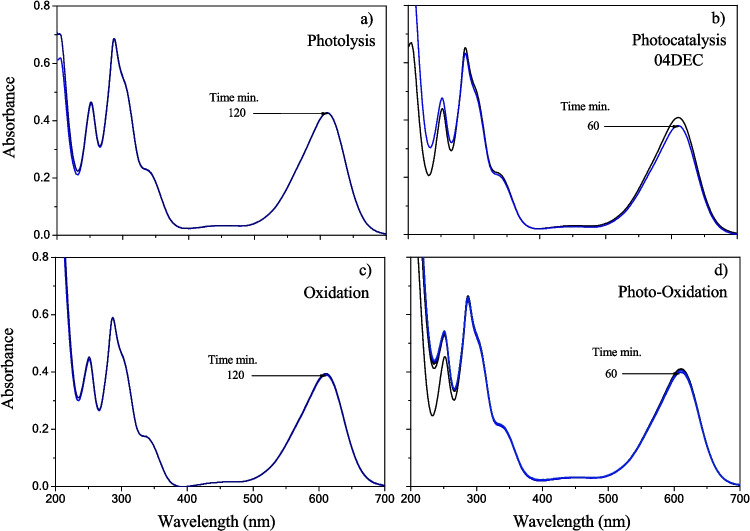
Absorbance spectra of 10 ppm of *IC* dye degradation as a function of UV light irradiation time, blanks of **a** photolysis, **b** photocatalysis (04DEC), **c** oxidation, and **d** photo-oxidation

This section shows the *IC* treatment processes which include the photolysis and photocatalysis performed with UV-C light and the oxidation and photo-oxidation performed in the presence of hydrogen peroxide (Table 1). The absorbance spectrum of the *IC* dye solution owed to the indigoid group (610 nm) was unaltered (Fig. 7a), when the photolysis process under UV light was carried out as blank at pH =  ~ 6.5 for 2 h of reaction. Then, the photocatalytic activity for all the composite samples was performed in similar conditions. The absorbance spectra of the *IC* solution for all the composite samples as a function of the illumination time are presented in Fig. 7b and S3 a-e. It was observed that absorbance spectra of the *IC* dye remained nearly unaltered after 60 min of illumination, decreasing the *IC* concentration by only 3.2% for 04DEC samples. It suggests that the photoactivity of either composite is negligible.

The absorbance spectrum of the *IC* dye at 610 nm was also unaltered (less than 1%) when the oxidation process (containing hydrogen peroxide) was carried out under dark conditions for 2 h of reaction (Fig. 7c). A similar tendency was also obtained when the photo-oxidation reaction (in the presence of peroxide and UV light) was carried out for 60 min of contact time (Fig. 7d), suggesting an apparent degradation or decolorization of 4%. All the processes for the *IC* treatment presented in this section suggest that neither of them is effective for decoloring nor removing the *IC* dye in the solution.

#### Fenton and photo-Fenton processes

**Fig. 8:**
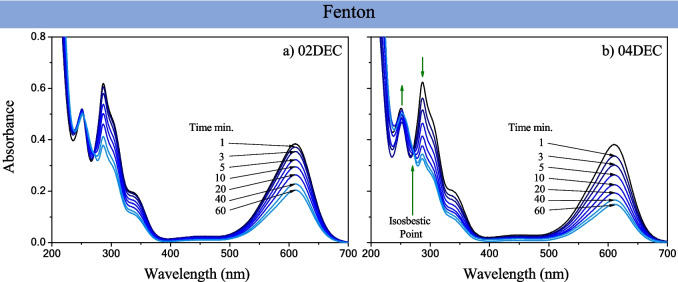
Absorbance spectra of 10 ppm of *IC* dye by applying the Fenton process in darkness as a function of time, using 5 mM of H_2_O_2_ and 0.1 mg/L of 02DEC and 04DEC samples at pH = 6.5

The Fenton activity of all the composite samples was evaluated in the degradation or discoloration of 10 ppm of *IC* dye with H_2_O_2_ in dark conditions. In the absence of composite materials, the oxidation reaction exhibited less than 1% of *IC* removal (Fig. 7c), and when the composite was added, an increase in the efficiency was detected. The absorbance bands of the *IC* dye at 610 and 287 nm decreased as time progressed in dark conditions (Fig. 8a–b and S4 a-d), suggesting a decrease in the *IC* dye concentration, which was more significant for the 04EC sample and for 04DEC, where more γ-Fe_2_O_3_ is formed. Furthermore, the diminution in the band at 610 and 287 nm was accompanied by an increase in the band at 243 nm, generating an isosbestic point at 251 nm. Analyzing the simulated theoretical spectra of *IC*, isatin sulfonic acid, and 2-amino-5-sulfobenzoic acid, computed with Time-Dependent Density Functional Theory (TD-DFT), reported by Hernández-Gordillo et al. 2019, the presence of an isosbestic point around 251 nm was identified, suggesting the presence of isatin sulfonic acid and 2-amino-5-sulfobenzoic acid as intermediates. This indicates that these two oxidation by-products are also formed when the Fenton process uses either composite because hydrogen peroxide induces the formation of hydroxyl or superoxide radicals. It is known that two possible routes can produce *IC* discoloration or degradation: the first by the formation of •OH radicals which can attack the C = C bond to produce isatin 5-sulfonic acid as the main aromatic product (Flox et al. 2006) and the second by the formation of superoxide radicals (•${O}_{2}^{-}$) that can also be produced by the Fenton process. Commonly, the Fe(II) is responsible for the production of •OH and as a result of this it is oxidized to Fe(III) to complete the Fe(III)/Fe(II) redox cycle Eq. (1), while the •${O}_{2}^{-}$ radicals are produced by the reduction of O_2_ and the disproportionation reaction of H_2_O_2_ due to the formation of Fe(III), following the sequential Eqs. (2–4) (Zeng et al. 2024):

1 $$Fe\left(II\right)+{H}_{2}{O}_{2}\to Fe\left(III\right)+\cdot \mathrm{OH}+{\mathrm{OH}}^{-}$$

2 $$Fe\left(II\right)+{O}_{2}+{e}^{-}\to Fe\left(III\right)+\cdot {O}_{2}^{-}$$

3 $$Fe\left(III\right)+{H}_{2}{O}_{2}\to Fe\left(III\right)+\cdot {O}_{2}^{-}+{2H}^{+}$$

4 $${HO}_{2}{\cdot }^{-}\to {H}^{+}+\cdot {O}_{2}^{-}$$

**Fig. 9:**
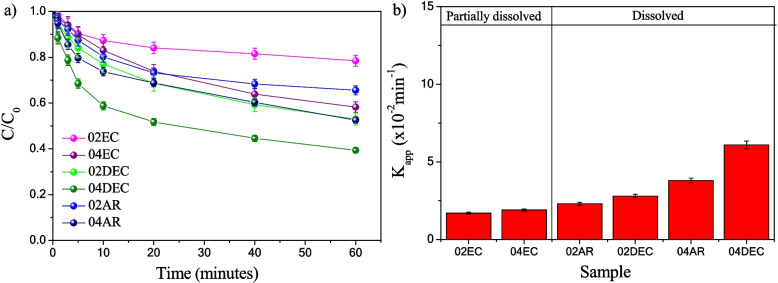
Plot of **a** *C*/*C*_0_ of *IC* dye in solution as a function of time and **b** apparent kinetic rate constant of *IC* dye by applying the Fenton method in darkness

Therefore, both •OH and •${O}_{2}^{-}$ radicals can be responsible for the *IC* discoloration into the above-mentioned two intermediates. Figure 9a shows the *IC* dye’s relative concentration profile (*C*/*C*_0_) versus time. The concentration profile exhibited a behavior of pseudo-first-order reaction. The calculated apparent kinetic rate constants (*K*_*app*_), obtained from the linear plot (S5a), are displayed in Fig. 9b with respect to composite materials. It is observed that the composite samples prepared with edible citric acid (02EC and 04EC) and a low concentration of analytical grade reagent 02AR exhibited low values of *K*_*app*_ (1.7 ± 0.1, 1.9 ± 0.1 and 2.3 ± 0.1 × 10^−2^ min^−1^, respectively). By contrast, the composite prepared with dissolved citric acid at 40 °C and with analytical grade reagent at 0.4 M showed the highest activity, with *K*_*app*_ values of 6.1 ± 0.2 and 3.7 ± 0.2 × 10^−2^ min^−1^ for 04DEC and 04AR, respectively.

**Fig. 10:**
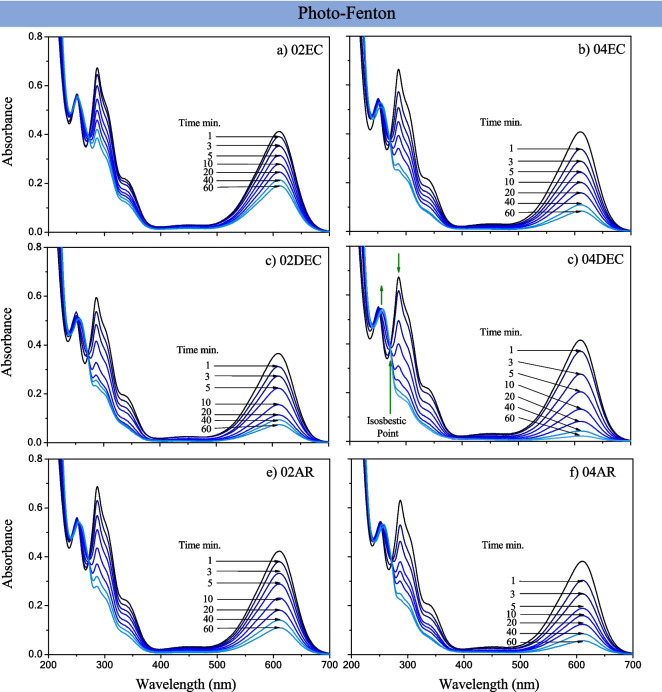
Absorbance spectra of 10 ppm of *IC* dye photo-Fenton degradation as a function of UV light irradiation time, using 5 mM of H_2_O_2_ and 0.1 mg/L of the synthesized composite material prepared with different types of citric acid grade reagents: **a** 02EC, **b** 04EC, **c** 02DEC, **d** 04DEC, **e** 02AR and **f** 04AR

The photo-Fenton activity of all the composite materials was also evaluated in the *IC* dye solution in the presence of H_2_O_2_ and under UV (365 nm) irradiation. The photo-oxidation reaction in the absence of composite also exhibited negligible *IC* removal (Fig. 7d), and when the composite was added, the percentage of removal was also increased. In this case, the decreasing band at 610 nm is also observed as the irradiation time progressed (Fig. 10a–f); the decreasing band was more significant for the 04DEC sample, representing 96.1 ± 2.1% of degradation or discoloration. The isosbestic point at 251 nm is observed again, suggesting the presence of the same intermediate products or oxidation by-products formed as in the photo-Fenton process (*isatin sulfonic acid* and *2-amino-5-sulfobenzoic acid*).

**Fig. 11:**
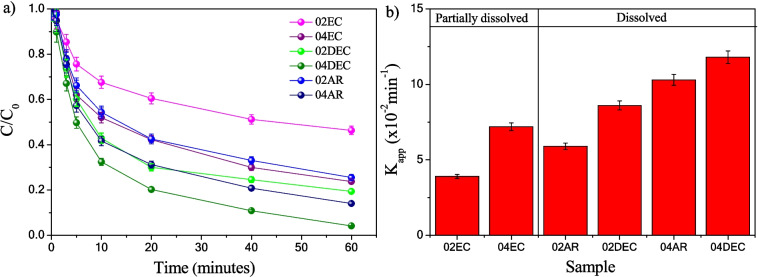
Plot of **a** C/C_0_ of *IC* dye in solution as a function of UV-light irradiation time and **b** apparent kinetic rate constant of *IC* dye photo-Fenton degradation using synthesized composite material prepared with different types of citric acid grade reagents: **a** EC, **b** DEC, and **c** AR at 0.2 and 0.4 M of citric acid

Figure 11a illustrates the *IC* dye (C/C_0_) degradation kinetics, which also exhibited a behavior of pseudo-first-order reaction: the calculated apparent kinetic rate constants (*K*_*app*_) are displayed in Fig. 11b. It is observed that composite samples prepared with edible citric acid (02EC and 04EC) and at low concentrations of analytical grade reagent 02AR exhibit the lowest *K*_*app*_ values of 3.9 ± 0.4, 7.2 ± 0.5, and 5.9 ± 0.7 × 10^−2^ min^−1^, respectively. By contrast, the composite prepared with citric acid dissolved at 40 °C (04DEC) and, with analytical grade reagent at 0.4 M (04AR), exhibited the highest activity, reaching *K*_*app*_ values of 11.8 ± 0.6 and 10.3 ± 0.8 × 10^−2^ min^−1^, respectively. These results can be explained because 02EC and 04EC have the lowest γ-Fe_2_O_3_ content (less than 61%), whereas 04DEC and 04AR contain 75.4 and 71.2% of γ-Fe_2_O_3_, respectively. Table 3 shows the standardized intrinsic *K*_*app*_ as a function of % γ-Fe_2_O_3_ (*K*_*app*_ × 10^−2^ min^−1^% γ-Fe_2_O_3_). As can be seen, the photo-Fenton process increased the intrinsic *K*_*app*_, presenting activity up to 1.9 to 3.7 times higher than the Fenton process.

**Table 3:** Degradation percentage and standardized intrinsic *K*_*app*_ as a function of % γ-Fe_2_O_3_ exhibited by all samples synthesized in the Fenton and photo-Fenton processes

Sample	Fenton		*Increasing*	Photo-Fenton	
	Degradation of *IC* (%)	*K*_*app*_ (× 10^−2^ min^−1^% γ-Fe_2_O_3_)		Degradation of *IC* (%)	*K*_*app*_ (× 10^−2^ min^−1^% γ-Fe_2_O_3_)
Partially dissolved citric acid					
02EC	21.4 ± 2.3	3.7 ± 0.1	2.3	53.6 ± 1.8	8.6 ± 0.4
04EC	41.8 ± 2.2	3.2 ± 0.1	3.7	75.8 ± 1.0	11.8 ± 0.6
Completely dissolved citric acid					
02DEC	46.6 ± 2.6	4.3 ± 0.1	3.1	80.0 ± 1.1	13.2 ± 0.5
**04DEC**	**60.8** ± 1.2	**8.1** ± 0.2	1.9	**96.1** ± 2.1	**15.6** ± 0.6
02AR	34.3 ± 1.4	4.2 ± 0.1	2.6	74.3 ± 0.9	10.7 ± 0.7
04AR	47.8 ± 1.3	5.3 ± 0.2	2.8	85.7 ± 0.8	14.5 ± 0.8

Data in bold emphasis indicate the material with the highest efficiency in the degradation of the indigo carmine dye

The photo-Fenton process was the most efficient process to carry out *IC* degradation using the composite as catalysts, containing nanoparticles of γ-Fe_2_O_3_ dispersed in a calcite matrix. The most active composite catalyst was the 04DEC material, with a degradation efficiency of 60 ± 1.2% and 96.1 ± 2.1% of *IC* in 60 min, in the Fenton and photo-Fenton processes, respectively. Comparing the results obtained by other authors, for the degradation of *IC*, most phases used are pure α-Fe_2_O_3_. However, they utilized analytical-grade reagents precursors to synthesize hematite (α-Fe_2_O_3_), and for the test photoactivity, powerful lamps with a large amount of photocatalyst material and an elevated H_2_O_2_ dose are typically used. For example, Yaou Balarabe et al. (2023) synthesized α-Fe_2_O_3_ sample was evaluated through the photo-Fenton process, and they managed to degrade around 65% of *IC* (25 ppm) in 35 min using a UV-light (125 watts Mercury lamp). Show et al. (2016) evaluated α-Fe_2_O_3_ nano-spheres through the photocatalysis process, degrading 96% of *IC* (100 ppm) in 150 min, using a 200 W tungsten lamp, obtaining a *K*_*app*_ of 1.9 × 10^−2^ min^−1^. In this work, the sample was synthesized from copper slag using edible grade citric acid to generate a copper slag lixiviate as a γ-Fe_2_O_3_ precursor. It was possible to degrade 96.1% of 10 ppm *IC* in 60 min, with a UV-lamp of 15 watts (less power than the reported works), 0.1 g/L, and 5 mM of H_2_0_2_, exhibiting *K*_*app*_ of 11.8 × 10^−2^ min^−1^.

**Fig. 12:**
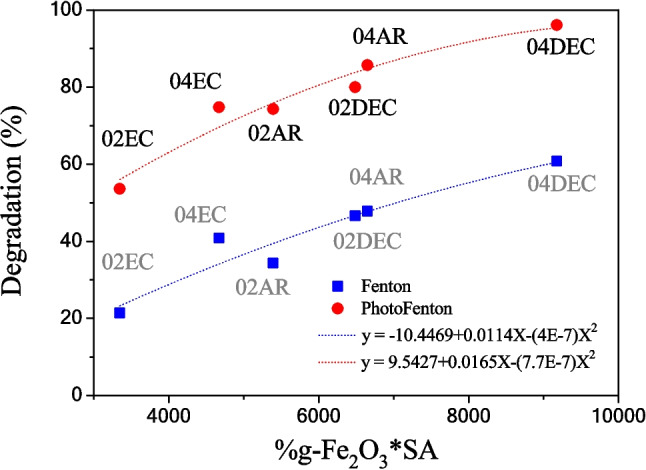
Plot of the degradation percentage of *IC* dye vs. the product γ-Fe_2_O_3_*SA in the Fenton and photo-Fenton processes

The photo-activity exhibited by composites in the photo-Fenton process could be related to diverse factors: (a) a higher content of the γ-Fe_2_O_3_ phase in the 04DEC sample (Table 3), (b) because γ-Fe_2_O_3_ nanoparticles are dispersed in the calcite matrix, and (c) a high SA. Additionally, metals such as Zn, Si, and Al that were leached from the CS sample when citric acid was added during the thermal treatment could produce ZnO, $\gamma$-Al_2_O_3_, and SiO_2_. However, the presence of these semiconductors cannot contribute to the generation of electron–hole pairs because the band gap values of the semiconductors ZnO, Al_2_O_3_, and SiO_2_ are 3.4, 7.0, and 9.0 eV, respectively, and thus, they cannot be excited at a wavelength of 365 nm (Jafarova and Orudzhev 2021; Khachaturova et al. 2022; Wang et al. 2025). Figure 12 illustrates the plot of the degradation percentage vs. the product % γ-Fe_2_O_3_*SA, where it can be seen that for Fenton and photo-Fenton processes, the greatest degradation percentage is achieved for the samples with a maximum value of this product (04DEC). In both Fenton and photo-Fenton processes, a second-degree polynomial is fitted, $y=-10.4469+0.0114X-(4\times {10}^{-7}){X}^{2}$ and $y=9.5427+0.0165X-(7.7\times {10}^{-7}){X}^{2}$, respectively. It is observed that the curve tends asymptotically to a maximum degradation of 100% with a limit likewise at the 100% of γ-Fe_2_O_3_ in the sample. This product % γ-Fe_2_O_3_*SA could provide a parametric factor to select samples with better removal capacities.

#### Determination of reactive oxygen species

**Fig. 13:**
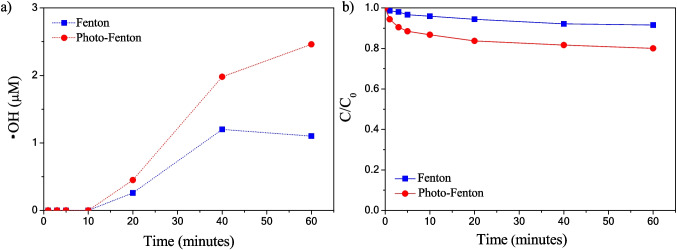
**a** Indirect quantification of hydroxyl radicals (•OH) applying salicylic acid dosimetry and **b** relative concentration *C*/*C*_0_ of *IC* dye in solution using 04DEC sample in anaerobic condition

In order to identify the radicals oxygen species (•OH and •${O}_{2}^{-}$) generated during the Fenton or photo-Fenton processes in the degradation of the *IC* dye, the formation of hydroxylated by-products, as indicative of the indirect generation of the •OH radical, was tested by applying salicylic acid dosimetry described in the “Determination of reactive oxygen species (ROS)” section. The sample 04DEC was used in the tests since it is the most efficient in the degradation of the *IC* dye. In the case of the Fenton process (Fig. 13a), a maximum concentration of 1.2 µM (0.19 mg/L) of •OH was quantified indirectly after 40 min of reaction, and a concentration of 1.0 µM (0.17 mg/L) of •OH after 60 min. For the photo-Fenton process, the maximum concentration of •OH radicals was 2.4 mM (0.38 mg/L) after 60 min. On the other hand, the formation of hydroxyterephthalic acid (HTA) measured by fluorescence was determined, as it can be observed in Fig. S6 a-b, where the band at 420 nm (HTA) grows, reaching a maximum between 20 and 40 min of reaction suggests indirectly an increasing in the production of •OH radicals for the Fenton process and photo-Fenton process at 60 min. Such behavior in the generation of •OH radicals agrees with that observed by salicylic acid dosimetry.

The formation of •${O}_{2}^{-}$ radicals using a photocatalytic test was evaluated with the 04DEC sample in anaerobic condition (absence of dissolved oxygen) to suppress the •${O}_{2}^{-}$ generation. After 60 min of photocatalytic reaction, the spectrum and the relative concentration *C*/*C*_0_ of *IC* remain almost unaltered (Fig. S7 and 13b). In the case of the Fenton process, the *IC* degradation was slowed to only 8.5% in anaerobic conditions, whereas in the photo-Fenton process, the *IC* degradation was slowed to 20% (Fig. 13). The absence of oxygen dissolved suppressed the •${O}_{2}^{-}$ generation and as a consequence decreased significantly the *IC* dye degradation, which suggests that •${O}_{2}^{-}$ radicals are primarily generated during the degradation process. However, •OH radicals are also generated in a smaller proportion than the •${O}_{2}^{-}$ radicals, which may be responsible for the 8.5 and 20% degradation of the *IC* dye by the Fenton and photo-Fenton processes in the absence of oxygen, respectively.

#### Stability of the sample and determination of iron leaching

**Fig. 14:**
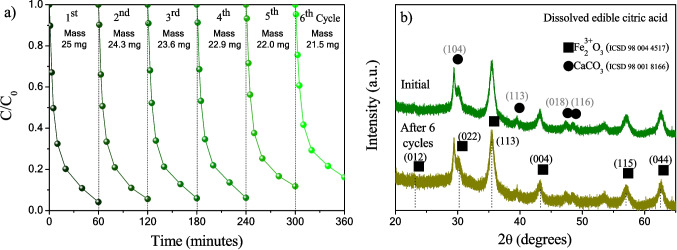
**a** Plot of C/C_0_ of *IC* dye in solution as a function of UV-light irradiation time for the 04DEC sample during the 6 cycles and **b** X-ray diffraction patterns of as-prepared and reused 04DEC sample (after 6 cycles of reaction)

Stability studies were performed using the most active composite sample (04DEC), tested during 6 cycles of reaction at 60 min of irradiation, under the same conditions for the photo-Fenton process (Fig. 14a). The sample exhibited 96.1% of *IC* degradation in the 1st cycle, maintaining its high activity up to the 4th cycle (95%); however, the activity begins to decrease for the 5 and 6th cycle, with a degradation of 85% in the 6th cycle. The mass loss at the end of the 4th cycle was 3 mg; therefore, the decrease in activity may be related to the loss of mass because the sample was trapped in the filter when each aliquot was taken. At the beginning of each cycle, the catalyst mass began to reduce; at the end of the 1st, 2nd, 3rd, 4th, and 5th cycles, 0.7, 0.7, 0.7, 0.9, and 0.5 mg were lost, respectively. These results indicate that the 04DEC sample presents excellent photostability during the photo-Fenton process recycled at less 4th cycle. In addition, the total iron leached measured by the photometric method during 6 cycles of reaction at 60 min of irradiation was $\le$ 0.03 mg/L, which is very low, indicating that the γ-Fe_2_O_3_ from 04DEC sample is stable during the photo-Fenton process (Fig. S8). In Fig. 14b, the diffraction pattern of the as-prepared and reused (after six reaction cycles) 04DEC sample is shown; a similar diffraction pattern with no significant changes is observed in the diffraction peaks at 23.5, 30.3, 35.6, 43.3, 57.2, and 62.9° in 2*θ*, corresponding to the (012), (022), (113), (004), (115), and (044) diffraction planes, respectively, which are associated with maghemite (γ-Fe_2_O_3_) (ICSD 98 004 4517), and other peaks are located at 29.4, 39.4, 47.6, and 48.5° in 2*θ*, corresponding to the (104), (113), (018), and (116) diffraction planes, respectively, which are associated with the calcite (CaCO_3_) (ICSD 98 001 8166). These results indicate that the reused 04DEC sample is chemically stable, and therefore, the decrease in activity of the reused sample during the photo-Fenton cycles process is due to the mass loss of the catalyst instead of the chemical instability of the material.

## Conclusions

Six composite catalysts with nano maghemite particles were successfully prepared from the lixiviate of copper slag using edible-grade citric acid at room temperature or at 40 °C and analytical-grade reagent citric acid at room temperature. The type of citric acid had an important influence on the maghemite (γ-Fe_2_O_3_) content of each catalyst, in the band gap, and in the specific surface area of these materials. The 04DEC sample exhibited the highest amount of γ-Fe_2_O_3_ (75.4%) and the highest specific surface area (121.7 m^2^ g^−1^), which is well-dispersed in a calcite matrix. The use of 0.1 g/L of composite in the photo-Fenton process degraded 96.1 ± 2.1% of the *IC* dye (10 ppm) in 60 min, with a UV-lamp of 15 Watts irradiation and 5 mM (100 µL) of H_2_O_2_; the degradation followed pseudo-first-order kinetics exhibiting *K*_*app*_ of 11.8 ± 0.6 × 10^−2^ min^−1^ for the 04DEC sample. The •${O}_{2}^{-}$ radicals are primarily generated during the degradation process; however, •OH radicals are also generated in a smaller proportion with respect •${O}_{2}^{-}$ radicals. The 04DEC sample exhibited 96.1% degradation of *IC* in the 1st cycle, maintaining high activity up to the 4th cycle. However, the activity began to decrease in the 5 and 6th cycle, with a degradation of 85% in the 6th cycle. The decrease in activity may be related to mass loss, as the sample trapped in the filter each aliquot was taken. This result is similar to the one reported by other authors who utilized pure maghemite synthesized using analytical grade reagents, as well as a higher dose of hydrogen peroxide during the Fenton or photo-Fenton process and a more powerful light source (125–200 W) in the photo-Fenton process. This suggests that it is feasible to use edible grade citric acid to generate *CS* leachates, which can be good precursors for obtaining a composite maghemite material on a nanometric scale, with a possible good cost–benefit ratio, which was very efficient for removing *IC* dye from water through the photo-Fenton method.

## Supplementary Information

Below is the link to the electronic supplementary material.

Supplementary file1 (DOCX 581 KB)

## Data Availability

In addition to data included as electronic supplementary material, data supporting the results reported in this paper can be accessed or available on reasonable request.
