# Supplementary material for: Copper metallurgical slag as a sustainable precursor of iron oxide photocatalysts to remove indigo carmine dye from water using the photo-Fenton process
Source: Environ Sci Pollut Res Int. 2025 Feb 19;33(6):1802–19. doi: 10.1007/s11356-025-36072-5 (PMC12960410; doi:10.1007/s11356-025-36072-5)
Supplement: Supplementary file 1 — Supplementary file1 (DOCX 581 KB) [file 11356_2025_36072_MOESM1_ESM.docx]

**Copper metallurgical slag as a sustainable precursor of iron oxide photo catalysts to remove indigo carmine dye from water using the Photo-Fenton process.**

Environmental Science and Pollution Research (ESPR)

Karen Valencia G.^1^, Melisa Portilla-Sangabriel^1^, Agileo Hernández-Gordillo^2^_,_ Tania A. García-Mejía^1^ and Rosa-María Ramírez-Zamora^1^

^1^ Coordinación de Ingeniería Ambiental, Instituto de Ingeniería, Universidad Nacional Autónoma de México, Circuito Escolar s/n, Ciudad Universitaria, 04510 Coyoacán, Ciudad de México, México.

^2^ Instituto de Investigaciones en Materiales, Universidad Nacional Autónoma de México, Circuito Exterior s/n, Ciudad Universitaria, 04510 Coyoacán, Ciudad de México, México.

Corresponding author email address: [RRamirezZ@iingen.unam.mx](mailto:RRamirezZ@iingen.unam.mx)

***S1*.** Nitrogen adsorption-desorption isotherm graphs were obtained from the BET surface area measurements of *CS*.

***S2*.** Nitrogen adsorption-desorption isotherm graphs obtained from BET surface area measurements of the synthesized composite material prepared with different types of citric acid grade reagents: a) EC, b) DEC, and c) AR at 0.2 and 0.4 M of citric acid.

***S3*.** Absorbance spectra of 10 ppm of *IC* dye degradation as a function of UV light irradiation time of Photocatalysis: a) 02EC, b) 04EC, c) 02DEC, d)02AR and e) 04AR.

***S4*.** Absorbance spectra of 10 ppm of *IC* dye by applying the Fenton process in darkness as a function of time, using 5mM of H_2_O_2_ and 0.1 g/L of a) 02EC, b) 04EC, c) 02AR and d) 04AR samples at pH = 6.5.

***S5*.** Pseudo-first order kinetics curves of *IC* dye degradation by applying the Fenton process in darkness as a function of time.

***S6*.** Fluorescence of hydroxyterephthalic acid (HTA) due to the production of •OH radicals using the 04DEC sample in the a) Fenton and b) photo-Fenton process.

***S7*.** Photocatalysis test of 04DEC in anaerobic condition in the a) Fenton and b) photo-Fenton process.

***S8*.** Total iron leached from the 04DEC sample was measured by photometric method during six reaction cycles.
